# A259 PEDIATRIC ACUTE LIVER FAILURE: A RETROSPECTIVE CROSS-SECTIONAL STUDY FROM A TERTIARY CARE CENTER IN JORDAN

**DOI:** 10.1093/jcag/gwac036.259

**Published:** 2023-03-07

**Authors:** E Altamimi, A Al Sharie, Y Al Zu’bi, R Mahadin, R Mahadin, S Al-Faqih, F Abu-Ekteish

**Affiliations:** 1 Pediatric Department-Faculty of Medicine; 2 Faculty of Medicine , Jordan University of Science and Technology, Irbid, Jordan

## Abstract

**Background:**

Pediatric acute liver failure (PALF) is a rare, rapidly progressing and life threating condition associated with high mortality rate despite optimal medical management. The clinicopathological characteristics of PALF could harbor new emerging avenues in patients’ stratification and management. Although, PALF is being studied extensively, no data from a Mediterranean cohort were elucidated in the medical literature

**Purpose:**

To analyse the clinical presentation, aetiology, and outcome of children presenting with acute liver failure, admitted to a tertiary hospital (King Abdullah University Hospital, Irbid, Jordan).

**Method:**

The electronic medical records at our center were retroactively accessed to identify PALF patients. Records of all children with acute liver failure over a 5-year period were reviewed retrospectively. Data on age, sex, clinical presentation, underlying cause, medical management, complication development and outcome were collected.

**Result(s):**

A total of 86 patients were identified, the mean age is 6.64 ± 5.82 years with male predominance (n = 46, 56.98%). The majority of PALF cases were not labeled with a definitive clinical diagnosis (n = 41, 47.67%). PALF etiologies include infections (n = 15, 17.44%), trauma (n = 10, 11.63%), malignancy-related (n = 9, 10.47%), genetic/metabolic (n = 7, 8.14%), and congenital anomalies (n = 4, 4.65%). The overall mortality rate is 43.02% and the average length of stay is 20.67 days. The majority of patients were managed in intensive care units (n = 67, 77.91%) and 20.93% (n = 18) had a positive blood culture during their hospitalization period.

**Conclusion(s):**

A more robust diagnostic algorithms should be implemented in PALF cases. The establishment of a country-based liver transplantation program is highly endorsed besides the implementation of many diagnostic tools to undercover the indeterminate causes of PALF.

**Please acknowledge all funding agencies by checking the applicable boxes below:**

None

**Disclosure of Interest:**

None Declared

